# MDCT Imaging Findings of Liver Cirrhosis: Spectrum of Hepatic and Extrahepatic Abdominal Complications

**DOI:** 10.1155/2013/129396

**Published:** 2013-08-06

**Authors:** Guillermo P. Sangster, Carlos H. Previgliano, Mathieu Nader, Elisa Chwoschtschinsky, Maureen G. Heldmann

**Affiliations:** Department of Radiology, LSU Health Shreveport, 1501 Kings Highway, Shreveport, LA 71103, USA

## Abstract

Hepatic cirrhosis is the clinical and pathologic result of a multifactorial chronic liver injury. It is well known that cirrhosis is the origin of multiple extrahepatic abdominal complications and a markedly increased risk of hepatocellular carcinoma (HCC). This tumor is the sixth most common malignancy worldwide and the third most common cause of cancer related death. With the rising incidence of HCC worldwide, awareness of the evolution of cirrhotic nodules into malignancy is critical for an early detection and treatment. Adequate imaging protocol selection with dynamic multiphase Multidetector Computed Tomography (MDCT) and reformatted images is crucial to differentiate and categorize the hepatic nodular dysplasia. Knowledge of the typical and less common extrahepatic abdominal manifestations is essential for accurately assessing patients with known or suspected hepatic disease. The objective of this paper is to illustrate the imaging spectrum of intra- and extrahepatic abdominal manifestations of hepatic cirrhosis seen on MDCT.

## 1. Introduction 

Hepatic cirrhosis is the clinical and pathologic result of a multifactorial chronic liver injury characterized by extensive fibrosis and nodular regeneration replacing the normal liver parenchyma [1]. It is well known that cirrhosis is associated with a markedly increased risk of hepatocellular carcinoma (HCC), the sixth most common malignancy worldwide and third most common cause of cancer related death. The detection of hepatic malignancy in cirrhotic patients is a diagnostic challenge due to distortion of the hepatic architecture [2]. In this article we discuss and illustrate the wide spectrum of intra- and extrahepatic findings on Computed Tomography (CT) in patients with cirrhosis.

## 2. Hepatic Manifestations

**Figure 1:**
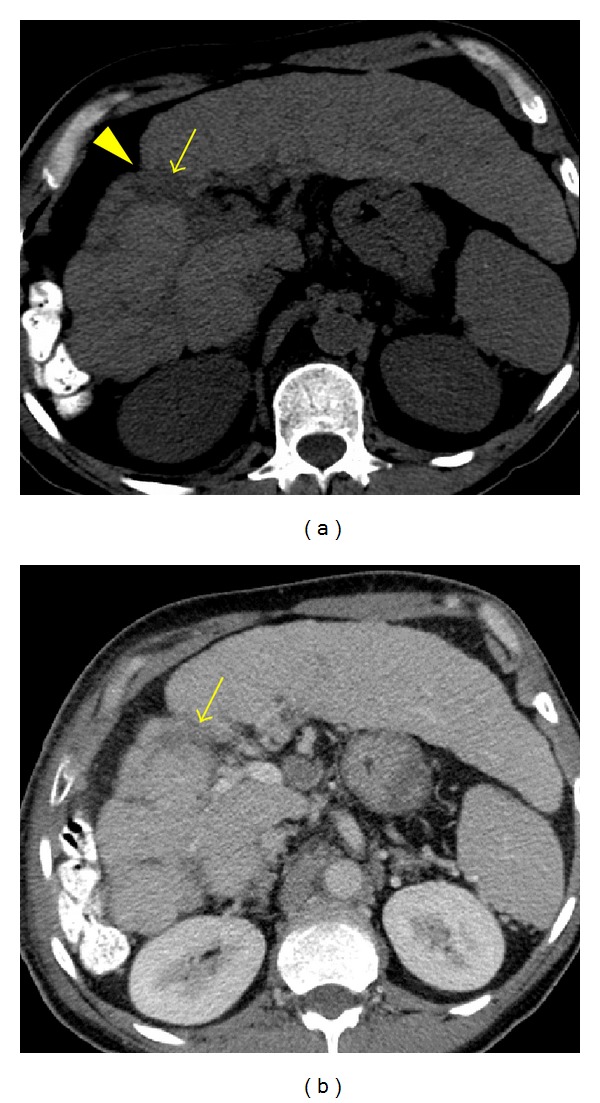
Confluent fibrosis in a 55-year-old male with alcoholic cirrhosis. (a) An unenhanced axial CT image shows a v-shaped area of subtle hypoattenuation (arrow) in hepatic segment 5. Note the retraction of the hepatic contour (arrowhead). (b) A portal venous phase axial image obtained at the same level as image (a) reveals an area of decreased portal venous flow (arrow).

Common pathologic features of cirrhosis include hepatic fibrosis, nodular distortion of hepatic architecture, and perfusion abnormalities. Thefibrotic changes appear as bridging bands or focal confluent fibrosis. Bridging bands usually have variable thickness and may mimic a tumor capsule due to delayed contrast enhancement. Focal confluent fibrosis is defined as a peripheral wedge-shaped hypoattenuated area on unenhanced and venous phase CT. On delayed phase, enhancement of the lesion may occur [3]. Overlying capsular retraction with volume loss in areas of focal confluent fibrosis is an important feature to differentiate this entity from malignant conditions [4] (Figure 1).

Morphologic changes of the liver vary with the stage of cirrhosis. More than 60% of patients with early cirrhosis have hepatomegaly. Additional early detectable morphologic changes of the liver include widening of the porta hepatis, enlargement of the interlobar fissure, and expansion of pericholecystic space [5]. During advanced stages shrinkage of the liver is seen, especially in alcohol-induced cirrhosis. The medial segment (IV) of the left lobe shrinks with concomitant hypertrophy of the lateral segments (II, III), giving a “tongue-like” appearance. These changes lead to a nodular contour and heterogeneity of the liver, which is classically associated with cirrhosis.

Hepatic steatosis is a nonspecific reversible response of hepatocytes to chronic injury, commonly seen in alcohol-induced cirrhosis. A diffuse uniform fatty infiltration involving the entire liver is the most common pattern. When hepatosteatosis occurs, the average liver attenuation is at least 10 Hounsfield Units (HU) less than the splenic parenchyma on unenhanced CT [6]. The identification of normal course vascular structures in areas of fatty infiltration is crucial to differentiate this abnormality from hepatic tumors. 

Evolving hepatic nodular lesions are another important feature of cirrhosis. In attempt to standardize the terminology, an international working party has suggested terms and definitions of nodular lesions in cirrhotic patients. These are categorized as regenerative nodules, dysplastic nodules, and HCC [7].

**Figure 2:**
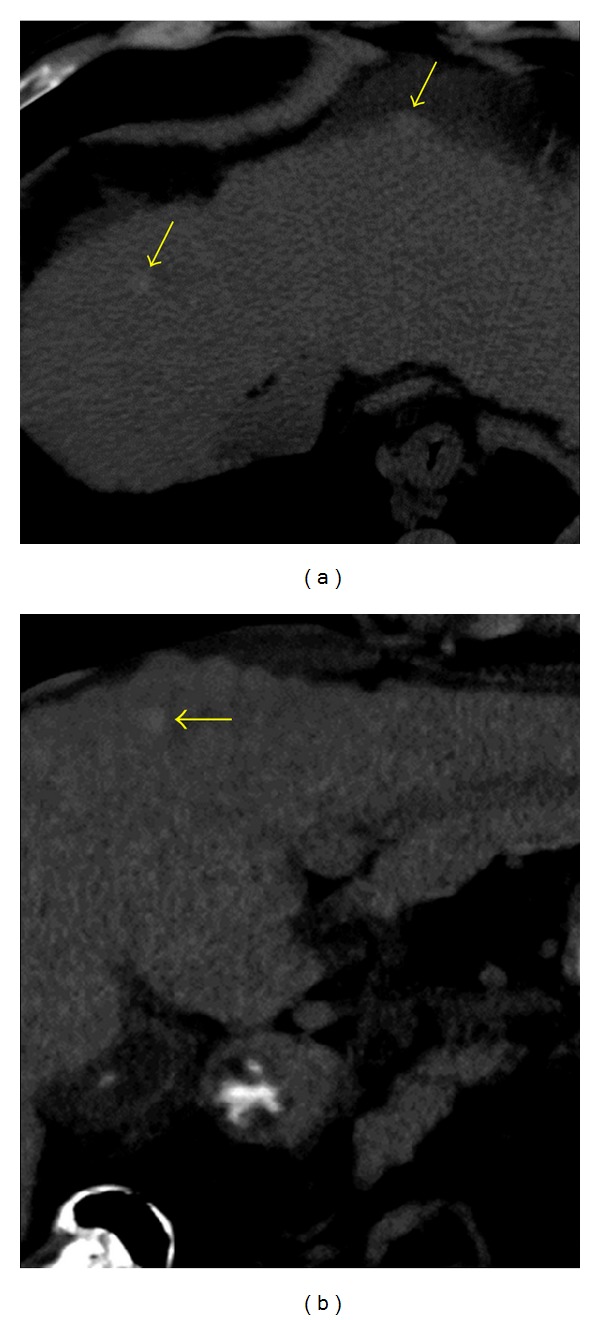
Siderotic regenerative nodules in a patient with cirrhosis. Unenhanced axial (a) and coronal (b) CT images show multiple subcentimeter high attenuation hepatic nodules (arrows). Note the nodular hepatic surface in this patient with micronodular cirrhosis.

**Figure 3:**
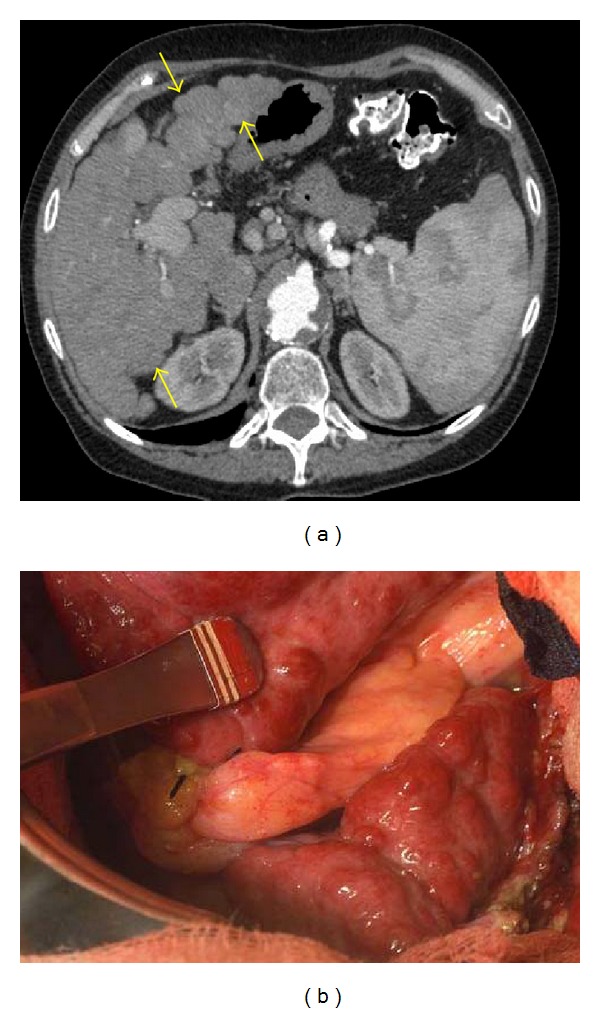
Macronodular regenerative nodules, due to alcohol-induced cirrhosis. (a) Arterial phase CT shows multiple nodular isodense lesions deforming the liver margin (arrows). The contour bulge caused by the nodular regeneration may help to detect the lesions. (b) Intraoperative photograph of macronodular cirrhosis.

A regenerative nodule (RN) is a well-defined area of liver parenchyma that has enlarged in response to necrosis and altered circulation. Based on gross morphologic features, the nodular regeneration can be classified as micronodular (<3 mm in diameter) or macronodular (>3 mm in diameter). Unless a regenerative nodule contains iron, it is rarely seen on a noncontrast CT [8]. If iron deposition is present (siderotic nodule), the nodule appears hyperdense to the surrounding liver on a non-contrast CT (Figure 2). Micronodular changes are rarely identified on CT, despite being present in all cirrhotic livers [8]. Regenerative nodules do not enhance in the arterial phase (Figure 3) and are isodense to the remaining parenchyma on the venous phase, making them indistinguishable from the hepatic background. The accuracy of non-contrast CT in detecting a RN is approximately 25% [8]. A combination of micro- and macronodular regeneration is the most common morphologic presentation seen in cirrhotic patients.

**Figure 4:**
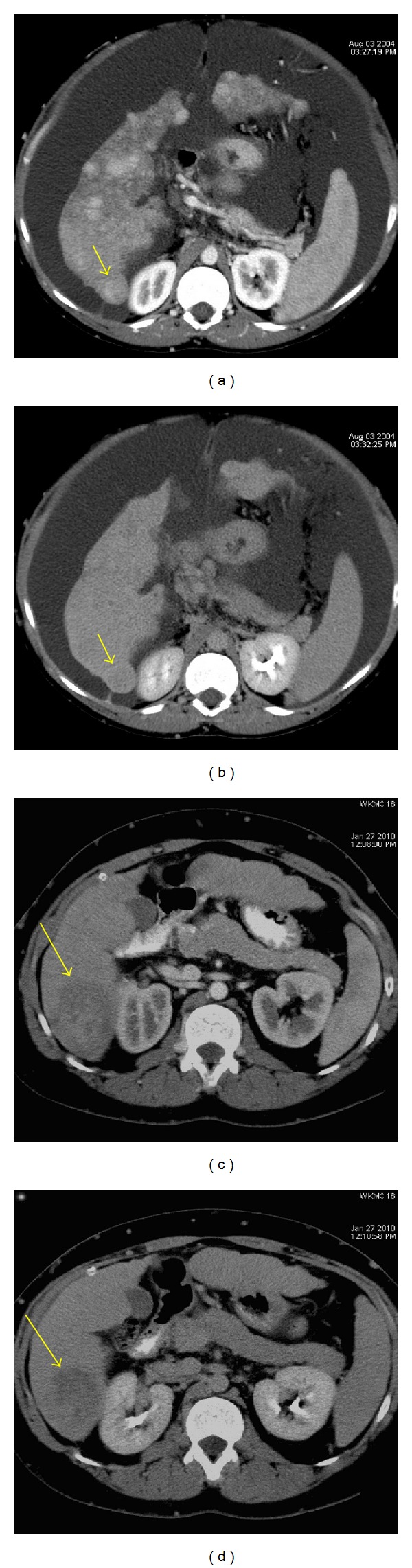
Dysplastic nodule in hepatic cirrhosis. Axial ((a), (b)) CT images during arterial and excretory phases show a dominant heterogeneous slightly hyperdense lesion in the segment VI compatible with dysplastic nodule (arrows); this lesion demonstrates a larger size compared with the remaining nodular hepatic lesions raising the possibility of DN in a patient with normal alpha feto protein value. Six-year imaging followup ((c), (d)) showing malignant transformation of this lesion (long arrows).

A dysplastic nodule (DN) is defined as a nodular region of dysplastic hepatocytes without histologic features of malignancy. DNs commonly measure 5–10 mm and most of them are undetectable by CT since, even after the administration of contrast, the majority is isoattenuating. Dysplastic nodules can be further characterized as low grade or high grade, according to the degree of dysplasia [7]. Tumor angiogenesis appears to be a mandatory step in the evolution of dysplastic nodules to HCC. During this process, there is a progressive increase in the arterial supply and a concomitant decrease in the portal venous supply to these lesions [9]. The major shift in angiogenesis typically occurs during the transition from low-grade DNs to high-grade DNs [10]. New vessels composed of nontriadal arteries become dominant and the absence of portal tracts is noted. The increasingly dedifferentiated nodule appears more markedly enhanced on post-contrast early arterial phase image, occasionally mimicking an HCC (Figure 4). Several reports have described the detectability of dysplastic nodules on dynamic CT scans. In a large series of liver transplantation specimens, small DNs (<5 mm) were never identified at preoperative imaging [9]. The detection rate for dysplastic nodules smaller than 2 cm has been reported, in pretransplant three-phase helical CT study, to be 39% [11].

HCC is a malignant neoplasm composed of cells with hepatocellular differentiation and is almost exclusively seen in patients with cirrhosis. The development of HCC in the cirrhotic liver is described either as de novo hepatocarcinogenesis or as a multistep progression, from low-grade dysplastic nodules to high-grade dysplastic nodule, then to dysplastic nodule with microscopic foci of HCC, then to small HCC, and finally to overt carcinoma [12]. 

HCC is classified histologically as trabecular, pseudoglandular, compact, and scirrhous, with the trabecular pattern being the most common. The fibrolamellar type of HCC has distinct clinical, histologic, and prognostic features and is commonly seen in young patients with no history of cirrhosis or chronic liver disease. The lesion appearance varies greatly according to size [13]. Small lesions enhance homogeneously, while large lesions are heterogeneous with a characteristic mosaic pattern, due to intralesional necrosis. Approximately 80%–90% of HCCs are highly vascular lesions demonstrating intense contrast enhancement during the arterial phase. In the venous phase, HCC demonstrates washout and becomes isodense with the liver parenchyma, thereby making its detection difficult [14]. About 10%–20% of HCCs are hypovascular and show contrast enhancement slightly less than that in the surrounding liver on arterial phase images, making the imaging differentiation with DNs difficult. The intranodular vascular changes of these lesions revealed by findings of CTAP (CT during arterial portography) and CTHA (CT during hepatic angiography) and correlated with histological analysis explain why high-grade DNs and early-stage-well-differentiated HCC are hypodense relative to the surrounding liver. Both lesions have decreased portal tracts (including normal hepatic arteries), without increased abnormal arteries. And, when the increased abnormal arterial supply compensated for the decreased normal hepatic arterial supply, they are isodense [15]. Understanding this blood supply pattern is important for early detection, characterization, and treatment for early-stage HCC [15].

**Figure 5:**
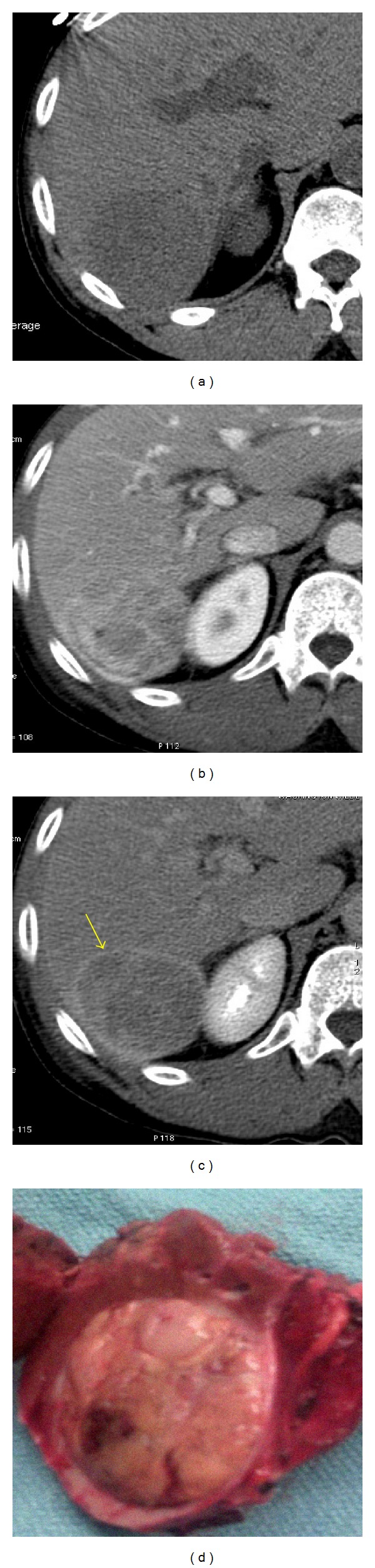
Solitary HCC. Axial CT images of the right hepatic lobe during precontrast (a), postcontrast venous (b), and delayed (c) phases show a well-defined heterogeneous solid enhancing mass occupying hepatic segment 7. Note the delayed enhancement of the lesion capsule ((c), arrow). (d) Photograph of the surgical specimen.

**Figure 6:**
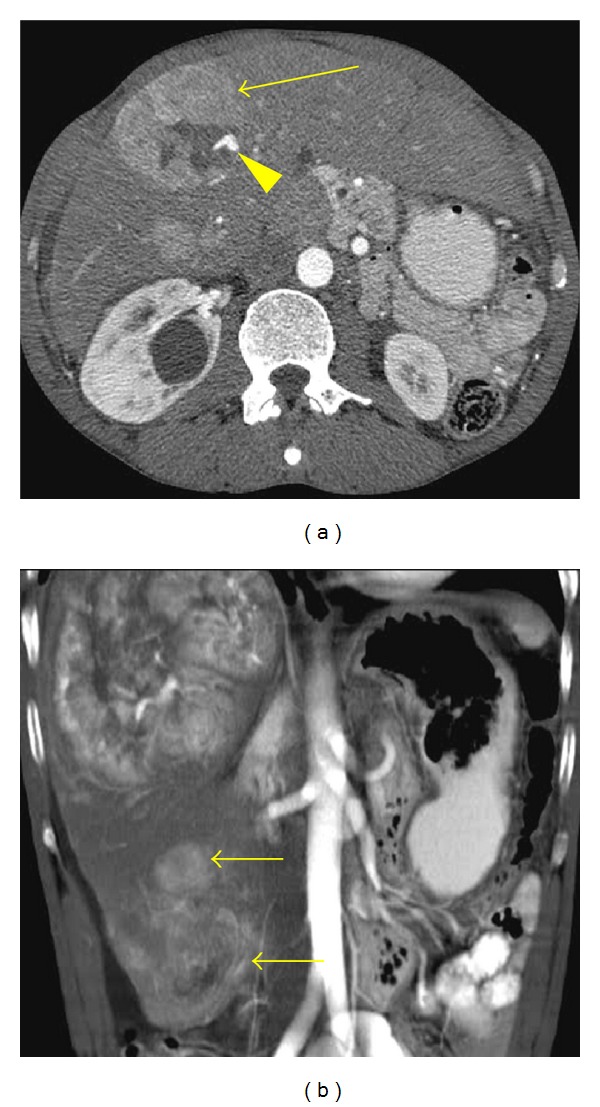
Multicentric HCC with a variegated appearance. (a) Arterial phase contrast-enhanced axial CT image shows a large heterogeneous mass that enhances intensely with multiple adjacent nodular areas with different attenuation patterns (long arrow). Intralesional arterioportal shunting is noted (arrowhead). (b) Coronal maximum intensity projection (MIP) reconstruction demonstrates additional smaller satellite hypervascular lesions (short arrows).

**Figure 7:**
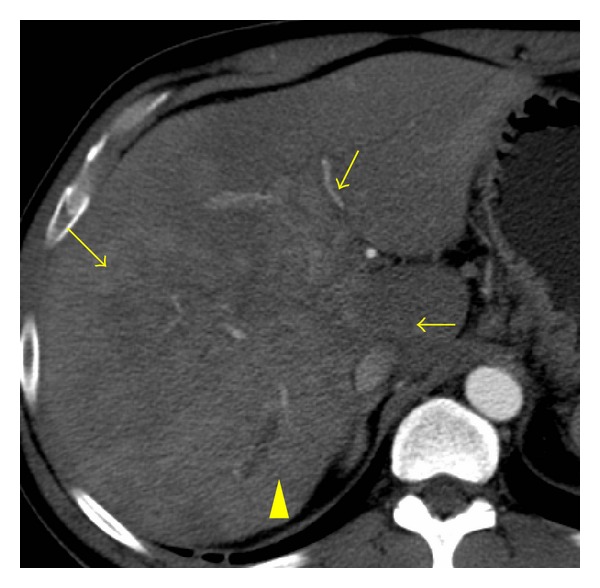
Diffuse hepatocellular carcinoma. Arterial-phase contrast enhanced axial CT scan demonstrates a large ill-defined heterogeneous mass occupying the right hepatic lobe (arrows). Focal intrahepatic biliary dilatation is seen (arrowhead).

**Figure 8:**
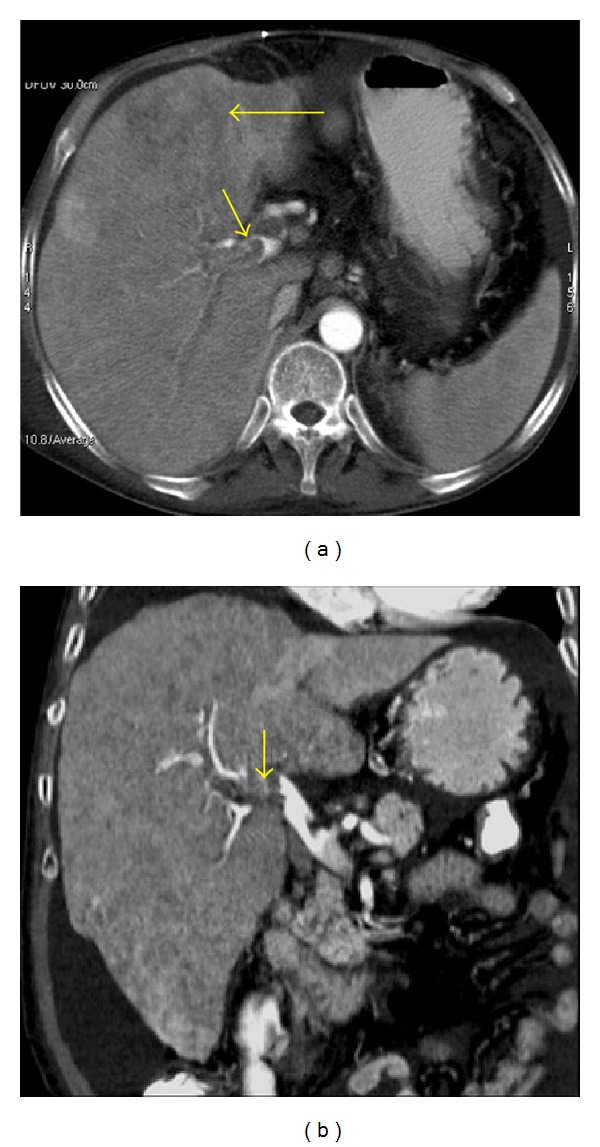
Portal vein thrombosis in a patient with HCC complicating hepatic cirrhosis. (a) Arterial phase contrast enhanced axial CT image shows a large filling defect in the portal vein indicating endoluminal thrombus (short arrow). A small peripheral HCC is noted in the hepatic segment 4 (long arrow). (b) a Coronal MIP reconstruction best depicts the filling defects in the main portal vein (arrow).

**Figure 9:**
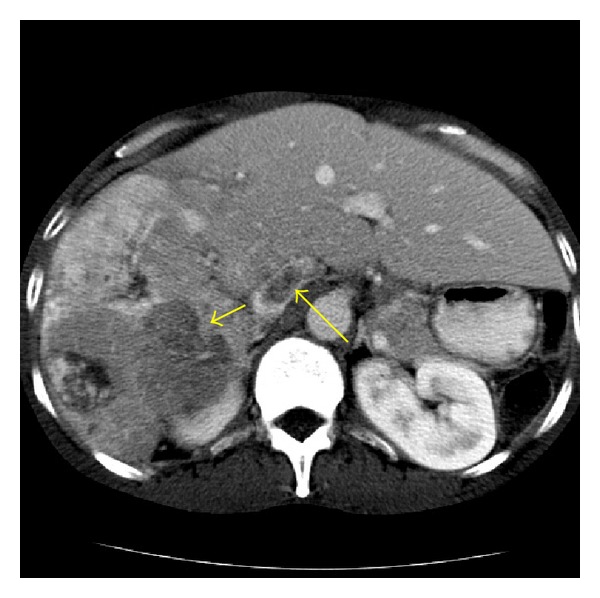
HCC invading the inferior vena cava. Contrast enhanced axial CT scan shows a central filling defect in the inferior vena cava (long arrow). Large peripheral enhancing hepatocellular carcinoma compressing the right kidney is also shown (short arrow).

Most HCCs have a fibrous capsule that is usually hypodense on hepatic arterial phase and enhance on delayed phase. HCC may present as a solitary mass (Figure 5), a dominant mass with daughter lesions (multicentric type) (Figure 6), or as a diffusely infiltrating neoplasm (Figure 7). Less frequently, it is multifocal with small foci usually less than 2 cm in both hepatic lobes, which may mimic liver metastasis [1]. HCC is very locally invasive and may extend to the bile ducts, portal vein (Figure 8), inferior vena cava (IVC) (Figure 9), and hepatic veins. Distant metastasis from HCC may be seen in the lungs, adrenals, adjacent lymph nodes, and bones. CT is accurate in staging HCC by detecting the number of lesions and involved segments, regional adenopathy, vascular tumor invasion, and metastases [14].

**Figure 10:**
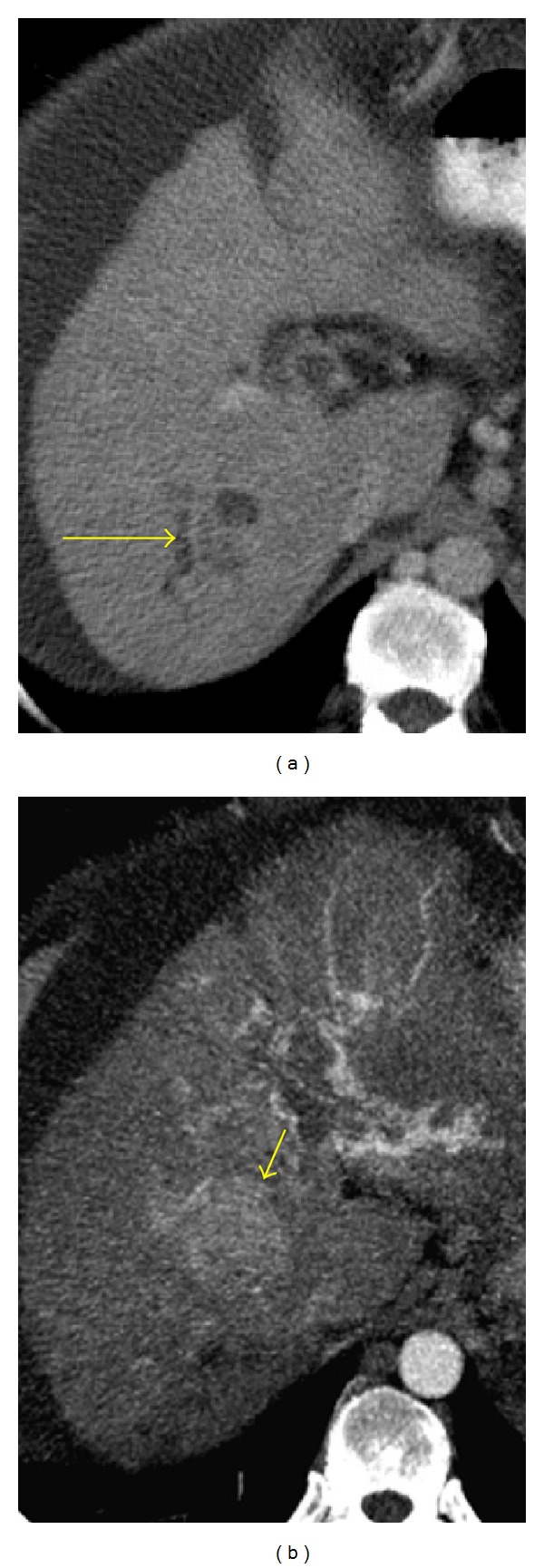
HCC only detected in the arterial phase. (a) A portal venous phase axial image shows intrahepatic biliary dilatation (long arrow) but fails to depict the HCC. (b) Arterial phase axial MIP reconstruction clearly delineates the hypervascular mass (short arrow).

The use of MDCT with dynamic contrast-enhanced triple-phase technique and reformatted images is essential to detect small HCC lesions; however, it remains the most challenging area in imaging cirrhotic liver. This technique demonstrates up to 30% more tumor nodules and in approximately 10% of cases of HCC will be the only phase to demonstrate the lesion [16] (Figure 10). Despite optimal arterial phase imaging, a large number of small (<1.5 cm) HCCs remain isodense relative to the background and go undetected on CT. Reported sensitivity for dynamic triple-phase contrast enhanced CT ranges from 50% to 96% and the specificity from 75% to 96%. It is well known that to obtain the best conspicuity of lesions, thinner slices (collimation, 1.5 mm; image reconstruction interval, 3 mm) and late arterial phase images (30–35 seconds after injection of contrast medium) should be acquired [17]. On the basis of explanted livers, it has been reported that the detection of hepatocellular carcinomas smaller than 2 cm, using three-phase helical dynamic CT, was 60% and the detection of those larger than 2 cm was 82% [11]. The reported sensitivity using previous state-of-the-art MR imaging technique and correlation with explanted liver pathologic results is also disappointing in detecting small HCCs and dysplastic nodules smaller than 2.0 cm [18]. 

Considering that in this country 60%–90% of HCCs occur in cirrhotic livers [19] and early-stage detection is difficult, the American Association for the Study of Liver Diseases (AASLD) includes a recommendation for periodic imaging surveillance in patients with liver cirrhosis and stated that the diagnosis of HCC can be made safe if a mass larger than 1.0 cm shows typical features of HCC (arterial hypervascularity and venous or delayed phase washout) at contrast material enhanced CT or MRI, obviating the need for biopsy if these features are present [20]. In our institution, surveillance guidelines are those based on the updated AASLD report. Four-phase MDCT is usually the preferred technique in our practice and contrast-enhanced MRI is used for problem solving cases or in those patients with any contraindication for contrast-enhanced CT.

**Figure 11:**
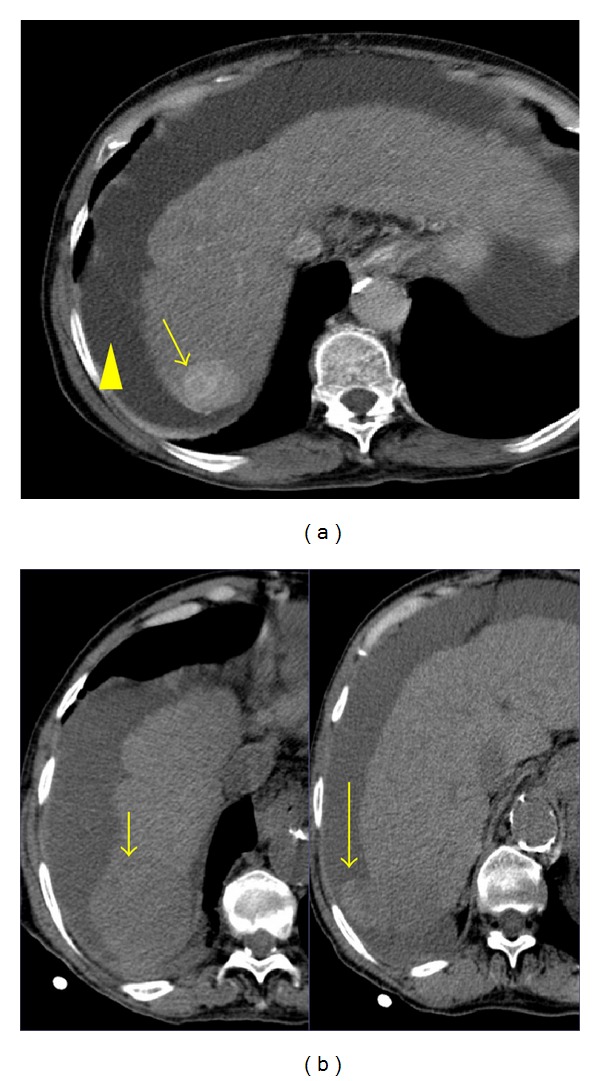
Ruptured HCC. (a) Contrast-enhanced CT shows a focus of HCC in the right lobe of the liver (long arrow). Low density fluid in the perihepatic space is also seen (arrowhead). (b) Three months later, the patient complained of increased abdominal pain. A nonenhanced CT obtained at the same level as (a) demonstrates an abnormally shaped right hepatic lobe (short arrow) associated with high density ascitic fluid consistent with hemoperitoneum (long arrow). Ruptured HCC was confirmed at surgery.

Arterioportal shunting is a well-known phenomenon that occurs in patients with cirrhosis and HCC. The typical imaging presentation is a wedge-shaped area of high attenuation seen on the arterial phase that becomes isodense to the liver parenchyma during the portal venous phase. The classic imaging finding is the presence of contrast material in distal portal branches, with minimal or no contrast in the proximal portal vein or superior mesenteric vein during the arterial phase [6]. Spontaneous rupture of HCC is an unusual complication identified in approximately 8% of the cases (Figure 11).

## 3. Extrahepatic Abdominal Manifestations

Extrahepatic abnormalities associated with cirrhosis include portal hypertension, ascites, splenomegaly, diffuse intra and retroperitoneal edema, small bowel, and gallbladder wall thickening. 

**Figure 12:**
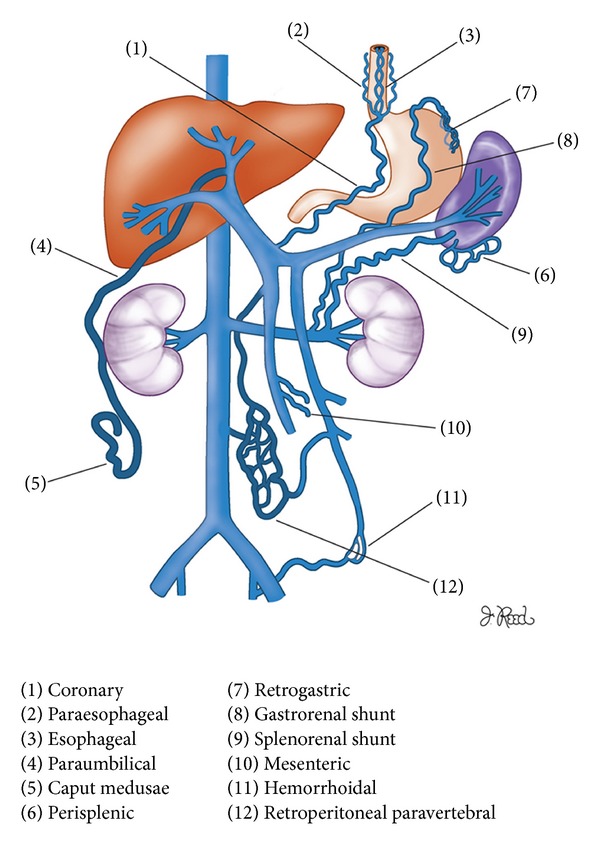
Portosystemic collateral vessels in portal hypertension.

A variety of morphologic alterations are seen in cirrhotic patients due to portal hypertension. Portal hypertension is defined as a portal pressure greater than 5–10 mm Hg. Portosystemic collaterals develop spontaneously, as blood flow is shunted away from the liver to low pressure systemic vessels (hepatofugal flow) [21] (Figure 12). Gastrointestinal variceal bleeding is the most common clinical presentation in patients with altered flow dynamics. 

Varices appear as well-defined tubular or serpentine homogeneous structures. On unenhanced CT, varices may mimic adenopathy, masses, or nonopacified bowel loops. The administration of intravenous contrast is vital to delineate dilated venous structures. 

The left gastric venous collaterals (coronary varices) are seen in approximately 80% of cirrhotic patients. These vessels are located in the lesser omentum, between the medial wall of the upper gastric body and the posterior margin of the left lobe. A 5-6 mm left gastric vein on CT is an indicator of portal hypertension. 

**Figure 13:**
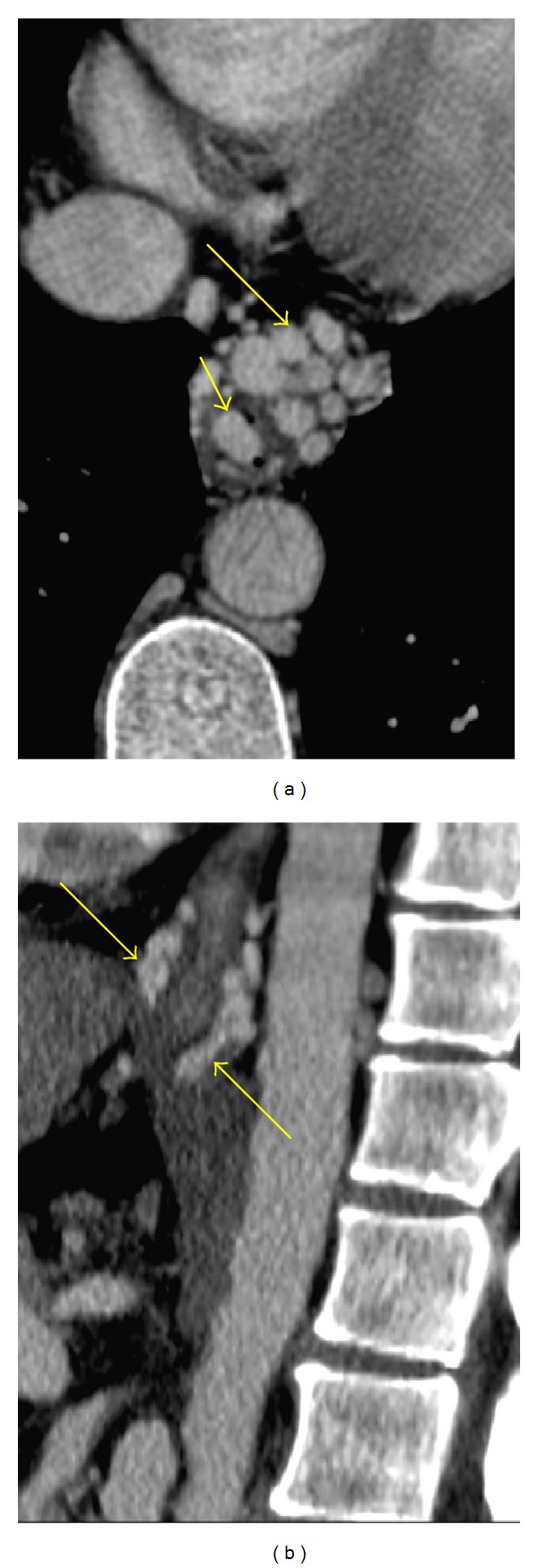
Esophageal and paraesophageal varices. Arterial phase axial (a) and sagittal (b) MIP reconstructions demonstrate multiple intramural (short arrow) and paraesophageal (long arrows) serpiginous tubular structures consistent with varices.

Esophageal varices are located in the wall of the lower esophagus and appear as intraluminal protrusions with scalloped borders (Figure 13). Paraesophageal varices are found around the esophageal wall and arise from the posterior branch of the left gastric vein, whereas the esophageal varices arise from the anterior branch.

**Figure 14:**
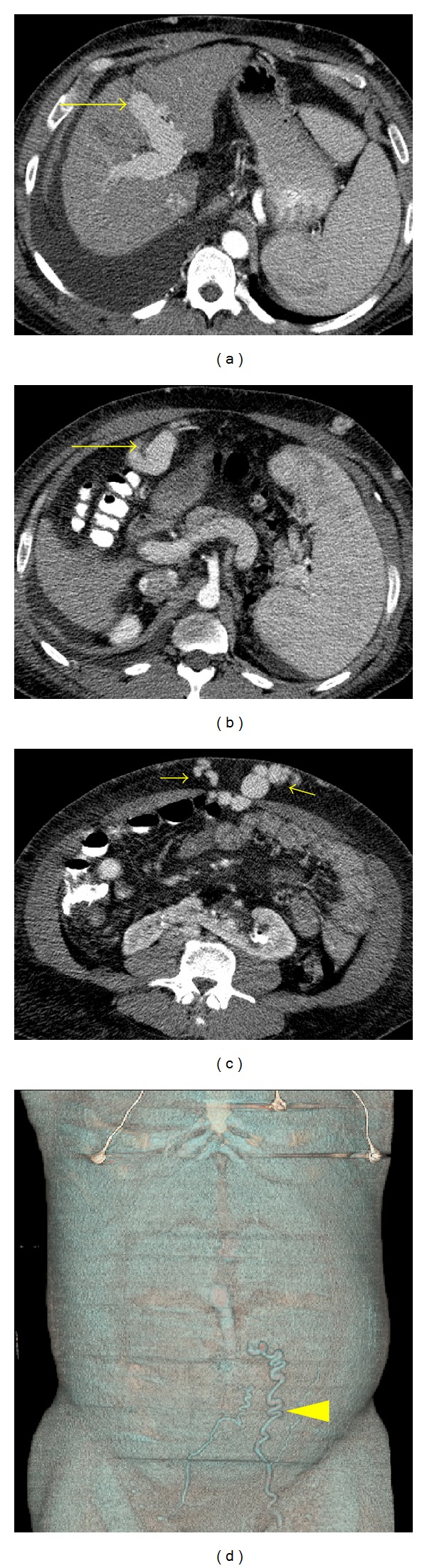
Paraumbilical varices. Contrast-enhanced axial images ((a)–(c)) demonstrate a paraumbilical varix originating from the left portal vein (long arrows) and extending to the abdominal wall (short arrows). A volume rendered reconstruction (d) demonstrates the caput medusae in the abdominal wall (arrow).

Paraumbilical venous collaterals vessels are found in 43% of patients with portal hypertension. They appear as circular or tubular enhancing structures in the falciform ligament and are supplied by the left portal vein. “Caput medusae” refers to collateral vessels that radiate from the umbilicus and are situated in the subcutaneous fat. These vessels are supplied by paraumbilical and omental veins (Figure 14). 

Another group of varices are seen in the anteroinferior aspect of the spleen. These varices are easily identified by their axial orientation and position in the perisplenic fat. 

The retrogastric varices are seen in the posteromedial aspect of the gastric fundus near the cardia and may be difficult to diagnose. They are fed by the left gastric or the gastroepiploic vein. 

**Figure 15:**
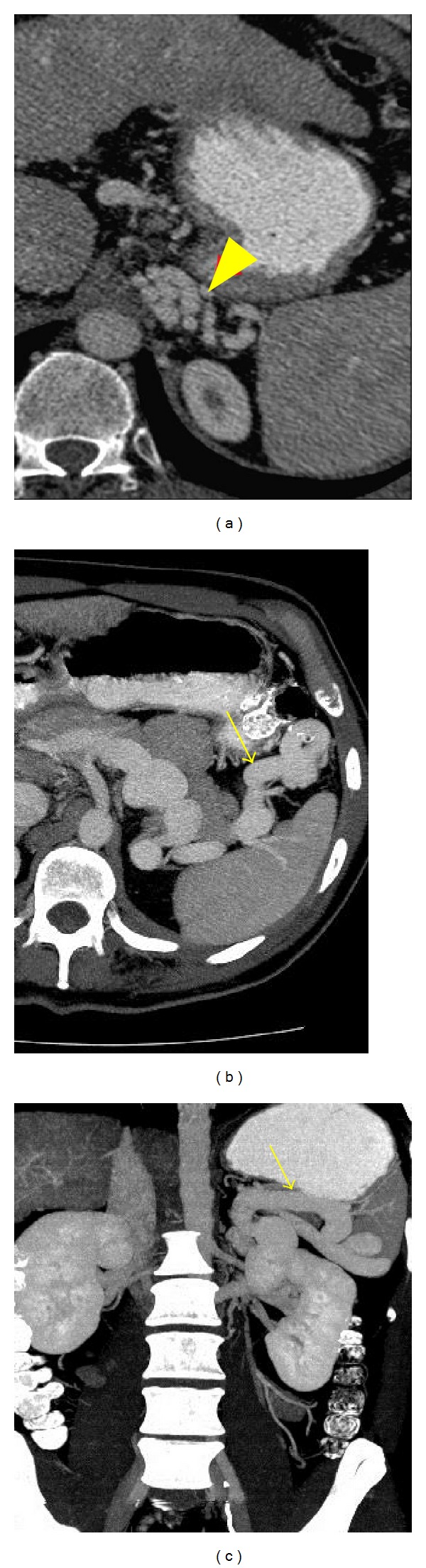
A cirrhotic patient with retrogastric varices and a splenorenal shunt. Axial contrast-enhanced images ((a), (b)) and a coronal MIP reconstruction (c) demonstrate retrogastric varices ((a), arrowhead) draining into a patent gastrorenal shunt (arrows).

Portosystemic shunts commonly involve the gastrorenal and the splenorenal systems. Retrogastric varices drain into the left renal vein through the gastrorenal shunt, whereas perisplenic varices drain directly into the left renal vein via the splenorenal shunt (Figure 15). 

**Figure 16:**
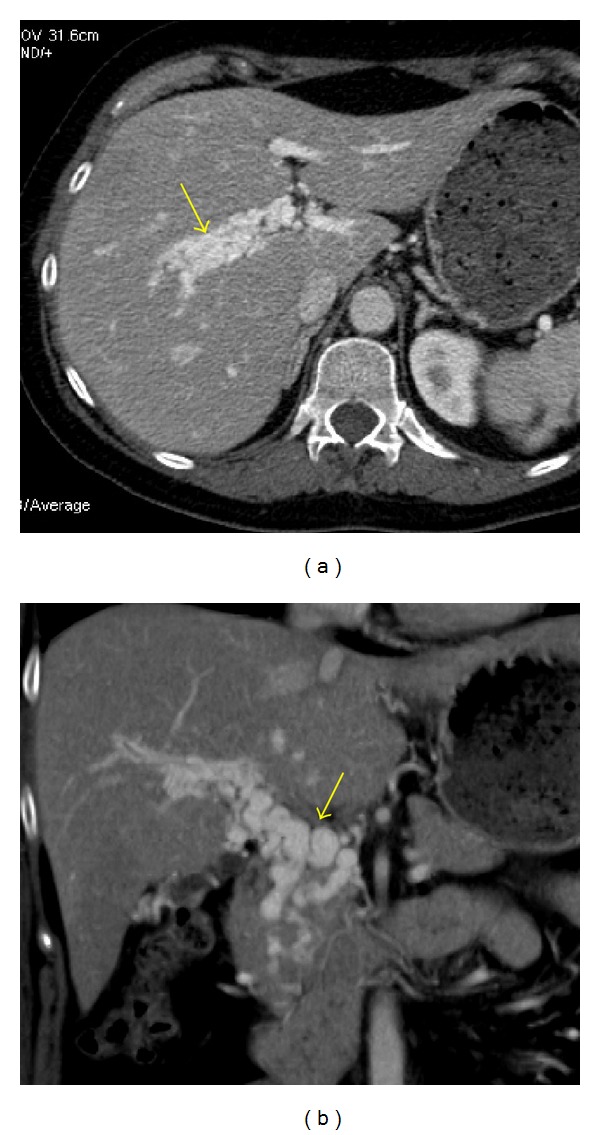
Chronic portal venous thrombosis with cavernous transformation of the portal vein. Axial (a) and coronal (b) contrast-enhanced CT images demonstrate multifocal serpiginous tubular structures in the hepatic hilum consistent with portoportal collateral vessels (arrows).

Portal vein thrombosis may occur in patients with cirrhosis and portal hypertension. After administration of contrast, the portal vein shows a central hypodensity corresponding to the intraluminal thrombus. In this situation, the hepatic arterial flow to the liver is increased, developing scattered peripheral transient high attenuation areas known as transient hepatic attenuation differences. In subacute and chronic portal thrombosis, a cavernous transformation of the portal vein may manifest as multiple tubular collaterals in the porta hepatis (Figure 16). When the portal vein is occupied by tumor thrombus, intraluminal enhancement may be seen.

**Figure 17:**
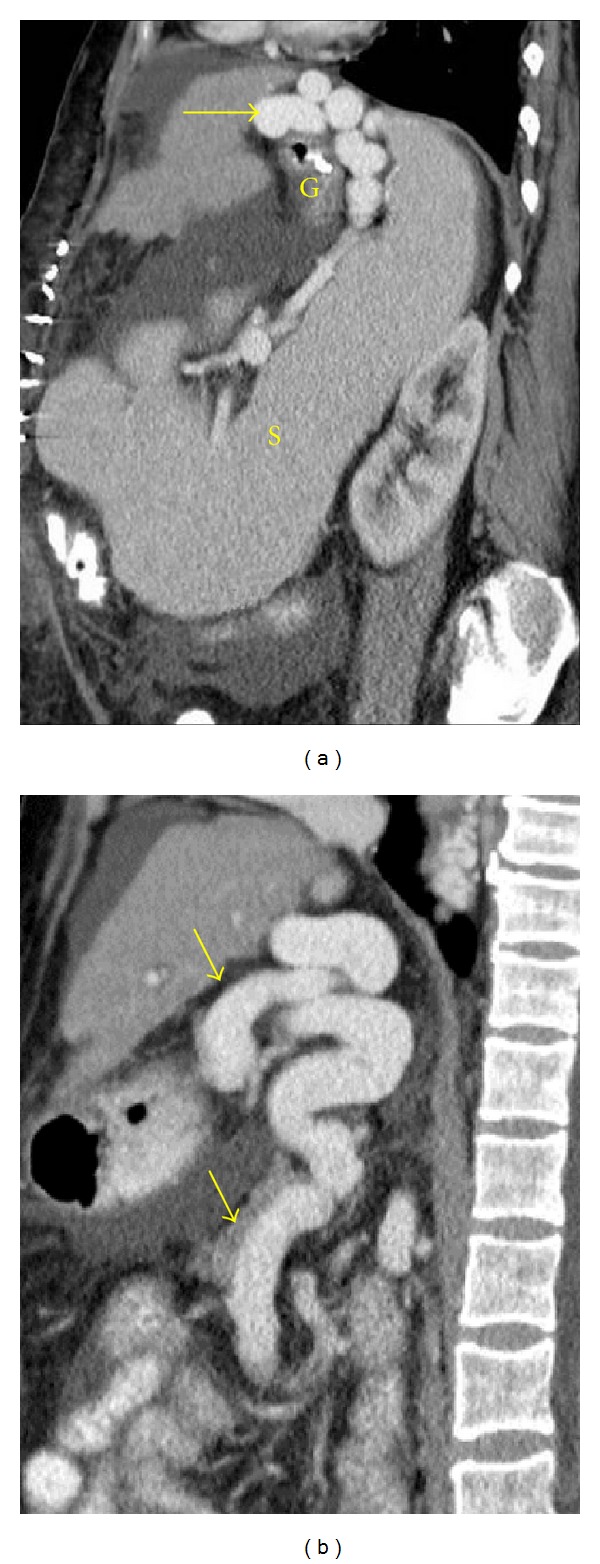
Splenomegaly in a cirrhotic patient with portal hypertension. Sagittal contrast-enhanced CT images ((a)-(b)) show an enlarged spleen (S) associated with retrogastric varices (long arrow) and splenorenal shunt (short arrows). G: gastric fundus.

**Figure 18:**
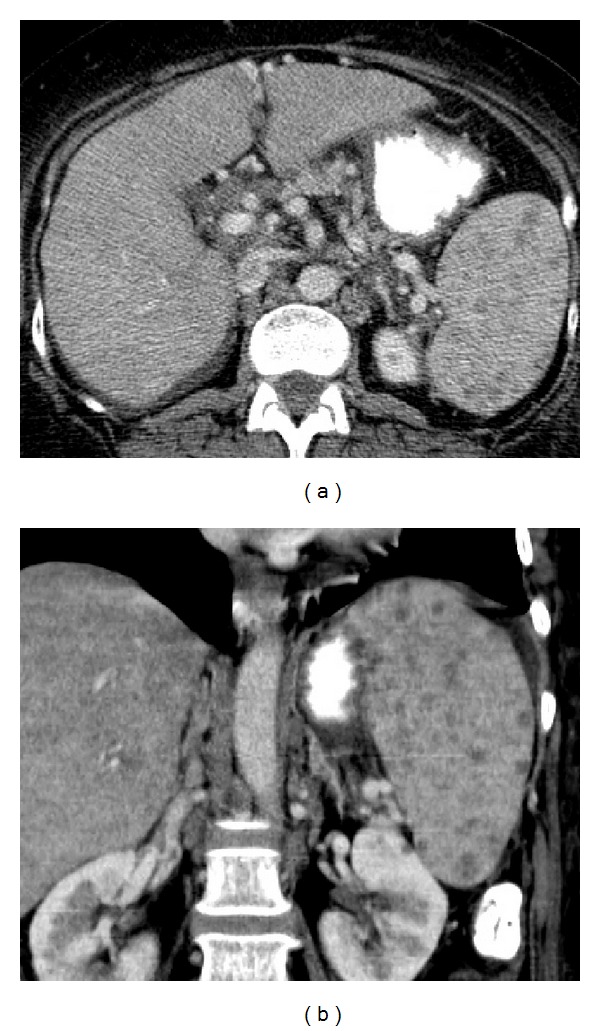
Gamna-Gandy bodies in a patient with portal hypertension. Axial (a) and coronal (b) contrast-enhanced CT images demonstrate an enlarged spleen with multiple low density foci representing hemosiderin deposition.

Portal hypertension is considered the most common cause of splenomegaly in the United States (Figure 17). Foci of hemosiderin deposition in the spleen are seen in about 9%–12% of patients with portal hypertension [22]. These foci are called Gamna-Gandy bodies, and their CT imaging pattern varies from hypo- to hyperdense spots, depending on the presence of secondary calcium deposition (Figure 18).

Mesenteric edema is defined as increased attenuation of the adipose tissue that surrounds the mesenteric vessels or their branches. Mesenteric edema in patients with cirrhosis has a multifactorial pathogenesis. Inflammation, hemorrhage, neoplastic infiltration, and hypoproteinemia due to hepatic insufficiency are the most frequent conditions identified. The frequency of mesenteric edema in patients with cirrhosis is 86%, and it is usually associated with omental and retroperitoneal edema. Most of the patients with mesenteric, omental, or retroperitoneal edema demonstrate a patchy, infiltrative pattern of fat stranding. The presence of retroperitoneal edema without mesenteric edema is uncommon. In some instances, focal edema may simulate a soft tissue mass. The severity of mesenteric edema parallels other manifestations of fluid overload in patients with cirrhosis such as subcutaneous edema, pleural effusion, and ascites [16].

**Figure 19:**
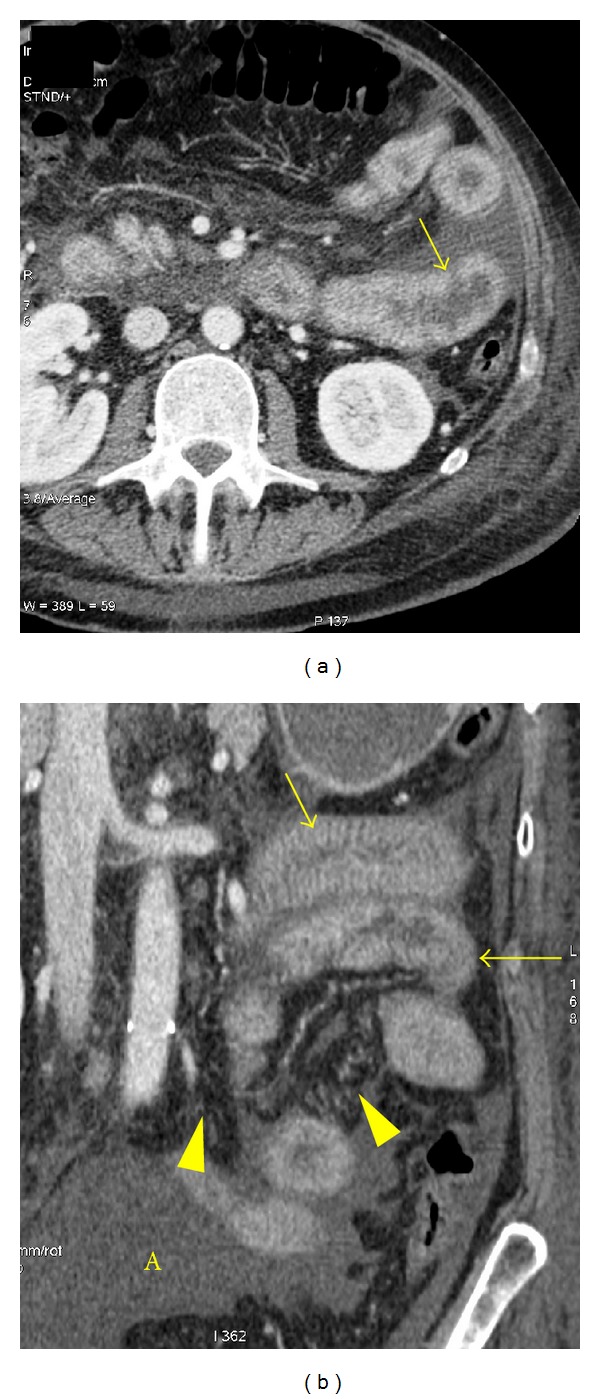
Small bowel wall thickening in a patient with hypoproteinemia due to hepatic cirrhosis. Axial (a) and coronal (b) contrast-enhanced CT images show diffuse thickening of the bowel wall and folds (arrows). Ascites (A) and mesenteric edema (arrowheads) are noted.

Gastrointestinal wall thickening occurs in 64% of cirrhotic patients, usually as a result of submucosal edema. The jejunum and the ascending colon are the most common sites of involvement (Figure 19). In almost all cases, the pattern of wall thickening is concentric with homogeneous enhancement after administration of intravenous contrast. Thickening of the colonic haustra has been described in patients with cirrhosis [17].

Hepatic cirrhosis may cause diffuse gallbladder wall thickening. The exact pathophysiologic mechanism leading to edema of the gallbladder wall is uncertain, but it is likely due to elevated portal venous pressure, decreased intravascular osmotic pressure, hypoproteinemia, or a combination of these factors [18]. Recognition of this abnormality is essential to avoid erroneous interpretations and unnecessary cholecystectomy. 

**Figure 20:**
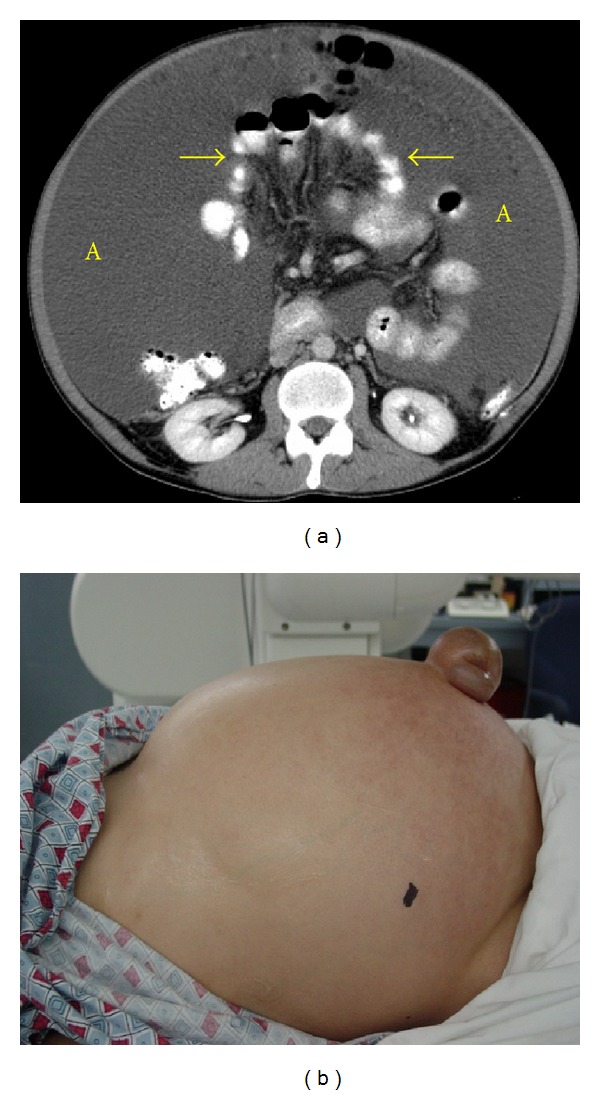
Tension ascites in a cirrhotic patient. (a) An axial contrast-enhanced CT demonstrates a large intraperitoneal low density fluid collection (A). Central displacement of the bowel and mesenteric structures (arrows) commonly seen in patients with benign intraperitoneal fluid (transudate). (b) Photograph of the patient's abdomen before decompressive paracentesis.

Ascites is defined as the pathologic accumulation of fluid in the peritoneal cavity. It is the most common complication of cirrhosis [19]. Within 10 years of the diagnosis of compensated cirrhosis, about 50% of patients will have developed ascites [20]. The development of ascites is the final consequence of anatomic and pathophysiologic abnormalities occurring in patients with cirrhosis. The formation of ascites is governed by the same principles as edema formation at other sites: net capillary permeability and the hydraulic/oncotic pressure gradients. Patients with cirrhosis but without portal hypertension do not develop ascites. Typical ascitic fluid in cirrhotic patients is a yellow-amber transudate with a total protein concentration of less than 2.5 g/dL and with relatively few cells (Figure 20).

## 4. Summary 

Hepatic cirrhosis is a multifactorial condition with increasing incidence worldwide. HCC, its most lethal complication, is seen in 11% of cases after 5 years of hepatitis related cirrhosis. A triple phase evaluation of the liver with CT is essential to detect small HCCs. The recognition of the extrahepatic abdominal complications is vital for adequate clinical assessment and treatment.
